# The Complex Relationship between Sleep and Cognitive Reserve: A Narrative Review Based on Human Studies

**DOI:** 10.3390/brainsci14070654

**Published:** 2024-06-27

**Authors:** Francesca Balsamo, Erica Berretta, Debora Meneo, Chiara Baglioni, Francesca Gelfo

**Affiliations:** 1 Department of Human Sciences, Guglielmo Marconi University, 00193 Rome, Italy; 2 IRCCS Fondazione Santa Lucia, 00179 Rome, Italy; 3 Department of Psychiatry and Psychotherapy, Faculty of Medicine, University of Freiburg, 79104 Freiburg, Germany

**Keywords:** sleep, cognitive reserve, cognitive functions, neuroprotection, humans

## Abstract

Sleep and brain/cognitive/neural reserve significantly impact well-being and cognition throughout life. This review aims to explore the intricate relationship between such factors, with reference to their effects on human cognitive functions. The specific goal is to understand the bidirectional influence that sleep and reserve exert on each other. Up to 6 February 2024, a methodical search of the literature was conducted using the PubMed database with terms related to brain, cognitive or neural reserve, and healthy or disturbed sleep. Based on the inclusion criteria, 11 articles were selected and analyzed for this review. The articles focus almost exclusively on cognitive reserve, with no explicit connection between sleep and brain or neural reserve. The results evidence sleep’s role as a builder of cognitive reserve and cognitive reserve’s role as a moderator in the effects of physiological and pathological sleep on cognitive functions. In conclusion, the findings of the present review support the notion that both sleep and cognitive reserve are critical factors in cognitive functioning. Deepening comprehension of the interactions between them is essential for devising strategies to enhance brain health and resilience against age- and pathology-related conditions.

## 1. Introduction

Genetic assets, prenatal- and postnatal environmental conditions, and life-experiences collectively contribute to determining individual differences and the distinctive manner in which each person lives, performs daily tasks, and confronts physiological aging and potential brain damage. 

Brain plasticity is the essential property of the nervous system through which brain structure and function vary in response to experience, purportedly enabling organisms to adapt to the surrounding environment [1,2,3]. Plasticity underlies learning and behavioral adaptation processes and allows compensatory modifications to occur in response to brain damage [3]. 

In this framework, the so-called cognitive reserve hypothesis [4,5] pertains to a complex architecture comprising various components, including brain, cognitive, and neural reserves. The term brain reserve (BR) refers to neuroanatomical and molecular features, ranging from brain size to synaptogenesis and neurotrophin expression [6], that promote resilience against aging and neural impairment [6,7,8,9,10]. Along with the concept of BR, the theoretical construct of cognitive reserve (CR) refers to the ability to effectively exploit the BR to perform specific tasks or maintain a certain cognitive function despite the presence of brain damage [5,9,10]. The conceptualization of CR started with the observation of interindividual variability in dementia-related clinical manifestations [4,5]. In fact, despite having nearly identical neurological damage, some individuals show delayed onset, slower progression, less severe symptoms, or better maintenance of premorbid functioning compared to others [4,5]. This variability can be attributed to differences in CR, indicating varying degrees of an individual’s ability to efficiently utilize neural networks, recruit alternative networks, or implement a different set of coping strategies to address tasks. Such ability results in compensating for the damage, thereby preserving (brain maintenance) or improving cognitive performance [11]. BR and CR have been described as the hardware (passive model) and software (active model) of reserve, respectively. However, this distinction overlooks the intimate connection between cognitive function and its biological substrate. Consequently, the term neural reserve (NR) has come into use to encompass both BR and CR [12,13]. 

BR and CR are built over time through a variety of plasticity-inducing experiences such as social, physical, and mental stimulations [14]. Experiences and lifestyle, including but not limited to education, work attainment, cognitive leisure activities, physical activity, sleep behaviors and attitudes, and diet, represent some of the so-called reserve builders that contribute to the BR and CR accumulation process (also referred as CR proxies) [1,5,14,15,16,17]. NR is dynamic by definition; it contributes to brain maintenance and can be used to face of age- or pathology-related brain insults [18]. Therefore, BR and CR constructs are key due to their clinical implications and potential for preventing age- and pathology-related clinical manifestations. Aging and neurodegeneration are progressive, incurable conditions; hence, it is imperative to gain a better understanding of NR components to maximize brain maintenance in patients and the elderly. 

Despite its well-demonstrated relationship with brain plasticity [19] and its impact on cognitive performance [20,21], sleep remains one of the most under-studied components of BR/CR/NR [15,22]. It is not entirely clear which features of sleep contributes to BR/CR/NR development, nor whether a robust BR/CR/NR protects against the effects of sleep disturbances on cognitive functions.

Sleep is a fundamental function shared by almost all animals [23]. The ubiquitous occurrence of alternating wake and sleep states across the animal kingdom has raised questions on the function of sleep. In fact, sleep is a costly activity, associated with increased vulnerability to predation [24]. There is a consensus that sleep serves a plethora of vital functions, including the immune response, energy balance, and development [25,26]. Sleep impairments in various species are associated with adverse effects on physiological functions, development, and cognitive abilities [23].

The timing, efficiency, and duration of sleep are regulated via a two-process model: a homeostatic process promoting sleep (process S) and a circadian process maintaining wakefulness (process C) [27]. The homeostatic drive for sleep (process S) depends on the length of preceding wake periods and is reflected in the electroencephalograph (EEG) power density of slow-wave activity (SWA) during Non-Rapid Eye Movement (NREM) sleep [28]. On the other hand, process C is independent of previous wake and sleep periods, representing the regulation of the endogenous circadian system upon sleep and wakefulness states [27]. In mammals, this system has a central pacemaker in the suprachiasmatic nucleus (SCN), which is synchronized with the external light–dark cycle [29]. In humans, the two processes consolidate into approximately a 16 h wake period during the day and an approximately 8 h sleep period during the night [30].

Excluding wakefulness, sleep can be grouped into two main phases: Rapid Eye Movement (REM) and NREM [31,32]. REM sleep is characterized by rapid eye movements, muscle atonia, rapid and low-voltage theta waves, and fluctuating heart and respiration rates [33,34]. In particular, it has been suggested that REM sleep plays a critical role in synaptic plasticity, facilitating learning and memory consolidation, and allowing the brain to replay and reorganize experiences [35,36,37]. These neuroprotective effects of REM sleep might be compromised in various sleep-related and neurodegenerative disorders [35].

NREM sleep comprises stage 1, stage 2, and slow-wave sleep (SWS), which is characterized by reduced neural activity and low-frequency, large-amplitude delta waves [33,34]. 

In pathological conditions, sleep regulation is altered due to different mechanisms. Sleep–wake disorders, as defined in major diagnostic manuals such as the Diagnostic and Statistical Manual of Mental Disorders [38] and the International Classification of Sleep Disorders [39], involve disturbances in the quality, timing, or duration of sleep that impact daytime functioning.

Insomnia disorder is a highly prevalent mental disorder associated with increased risk of developing other mental and somatic disorders [40,41,42] and high personal and societal costs [43,44]. It is characterized by persistent difficulty in initiating or maintaining sleep, or complaints of nonrestorative sleep, accompanied by daytime consequences in cognitive, affective, and functional domains [38,39].

Other sleep–wake disorders are more commonly due to physiological alterations. Sleep-related breathing disorders (SRBD), such as obstructive (OSA) and central (CSA) sleep apnea, are caused by altered physiological mechanisms that affect breathing during sleep [45]. 

Furthermore, other sleep disturbances, known as parasomnias, are characterized by unwanted behaviors and movements occurring during sleep or upon awakening [46]. Examples include REM sleep behavior disorder (RBD) [47,48] and isolated REM sleep behavior disorder (iRBD) [35,46,49], which can lead to the development of progressive neurodegenerative diseases [46,47,48].

The assessment of sleep–wake disorders is fundamental to prevent long-lasting negative consequences. Notably, in 2014 Buysse emphasized that good sleep health is not just the absence of a pathological condition. Poor sleep health can compromise overall quality of life, psychological wellbeing, and cognitive aspects, even in non-clinical populations [50]. Based on extensive literature, Buysse (2014) defined sleep health as a continuum from pathology to optimal health, in which five dimensions are particularly relevant: sleep duration (the amount of sleep in the 24 h period), sleep timing (the allocation of the major sleep episode in the 24 h period), sleep efficiency (the ratio of total time spent asleep to total time spent in bed), sleep satisfaction (the subjective satisfaction with one’s own sleep), and daytime alertness (the ability to maintain attentive wakefulness during the day) [50]. These dimensions are included due to the vast and robust literature underlining their association with health outcomes in children, adolescents, and adult populations [50,51]. Such negative outcomes include risk of obesity [52], all-cause mortality [53], cardiovascular disease [54,55], and metabolic syndrome [56]. Poor sleep health is also associated with a higher risk of mental health problems both during developmental years [57] and in adulthood [58]. 

The amount of sleep is the most extensively studied sleep dimension. It has been associated with emotional, cognitive, and somatic functioning, and several studies show how sleep deprivation impacts central nervous system (CNS) implicated in emotion regulation, attention, memory, energy balance, and hormonal secretion [59,60,61]. Nevertheless, additional dimensions of sleep health also play a crucial role in determining cognitive functioning throughout one’s lifespan (e.g., [42,50,51,62]). 

Therefore, both BR/CR/NR and sleep play critical roles in moderating brain function and influencing the clinical expression of cognition and behavior [8,12]. Thus, modifying behavior to promote healthy lifestyles can enhance CNS capacity to cope with pathological changes, mitigate the negative effects of both healthy and pathological aging, address sleep-related impairments, and protect against cognitive decline [7,11,15,63]. Nevertheless, the relationship between BR/CR/NR and sleep behaviors and disorders remains under-researched [15,63]. While several studies highlight the beneficial effect of sleep on cognitive performance [64,65,66,67], few specifically investigate the link between sleep and reserve, such as sleep’s role in supporting BR/CR/NR [15] or reserve’s impact on mitigating and improving sleep quality.

Given these gaps, the present literature review aims to explore the relationship between BR/CR/NR and sleep in humans within the context of cognitive functions. Specifically, it seeks to understand the bidirectional influence these factors exert on each other and examine how the existing scientific literature explicitly connects them.

## 2. Literature Search Strategy

A methodical search of the literature was conducted using the PubMed database to identify articles focusing on the interaction between two key areas of interest: sleep and BR/CR/NR. Although this is a narrative review, we qualitatively consider all available literature up to February 6, 2024, with no restriction on the publication date.

The search strategy involved terms related to BR/CR/NR and healthy or disturbed sleep. Specifically, the advanced search method used in the PubMed database was as follows: 

((“cognitive reserve” OR “brain reserve” OR “neural reserve”) AND (insomnia OR sleep OR “sleep disorder*” OR “healthy sleep*”)).

Only clinical studies written in English and involving human subjects were included, while grey literature was excluded.

**Figure 1:**
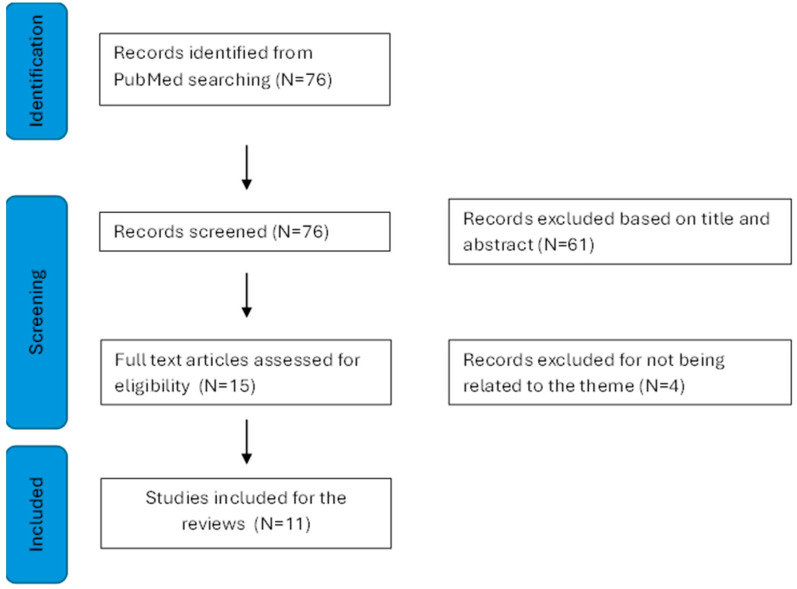
Presents a detailed flowchart of the literature search, conducted following the Preferred Reporting Items for Systematic Reviews and Meta-Analyses (PRISMA) statement [68].

This advanced search yielded a total of 76 articles. After screening titles and abstracts, 15 publications were initially considered. However, upon full-text examination, 4 publications were excluded for not meeting inclusion criteria, leaving 11 articles for the present review (see Figure 1).

The inclusion criteria were utilized to assess study eligibility and encompassed human subjects, comprising both healthy individuals and those with pathological conditions, without restrictions based on sex or age. Studies that explicitly addressed both sleep and BR/CR/NR were included, while those focusing solely on one of these elements were excluded.

For the purpose of this review, the relevant data considered consisted of the age and sex of human subjects, sample size, population (healthy/pathological), sleep component/disorder, cognitive function investigated, and method used to assess sleep, cognitive domains, and reserve (see Table 1, Table 2 and Table 3). 

## 3. Results

The literature review provides evidence on the topic. Out of the 11 scientific articles, 9 focused on CR as a variable that protects against sleep behavior/disorders in relation to cognitive function [69,70,71,72,73,74,75,76,77]. Two articles focused on sleep as a factor that enhances CR [22,78] (See Table 1). Instead, none of the articles included in the review explicitly addressed the concepts of BR and NR. 

These results will be outlined in two main sections, each organized according to a population-based logical structure:

1. The first section discusses findings related to sleep as a factor that enhances CR;
2. The second section discusses findings regarding CR as a moderator between sleep and cognitive functions.

### 3.1. Sleep as Factor Involved in Cognitive Reserve

Only two studies have examined the role of sleep as a factor involved in the modulation of CR [22,78]. Specifically, one study involved a healthy population [22], while the other study was conducted on a pathological population, particularly, individuals affected by Alzheimer’s disease (AD) [78] (see Table 2).

#### 3.1.1. Healthy Population

**Zijlmans and colleagues** [22] aimed to investigate whether objectively estimated sleep and 24 h activity rhythms, which may reflect the physiological aspect of sleep, are associated with CR.

To investigate this relationship, participants from a population-based cohort study (mean age: 65.0 years) underwent a comprehensive assessment of sleep over 7 consecutive days and nights using actigraphy, alongside maintaining a sleep diary. Additionally, they completed the Pittsburgh Sleep Quality Index Questionnaire (PSQI). CR was evaluated through a battery of cognitive tests, covering various cognitive domains such as verbal memory, attention and interference of automatic processes, long-term memory, processing speed, and fine motor skills (for more details see Table 2). Brain Magnetic Resonance Imaging (Brain-MRI) scans were performed to analyze brain volume. CR was operationalized as a latent variable encompassing the variability among cognitive scores, while controlling for age, gender, education, total brain volume, intracranial volume, and white matter hyperintensity volume. A higher CR score, indicating a more positive residual, reflected superior cognitive functioning compared to expectations based on current cognitive status, age, gender, education, total brain volume, white matter hyperintensity volume, and intracranial volume.

The relationship between sleep patterns, including 24 h activity rhythms, and CR was then explored using structural equation models. The findings revealed that prolonged sleep onset latency and reduced sleep efficiency correlated with diminished CR. Consequently, these results support the notion that sleep, in conjunction with other variables, plays a role in shaping CR levels [22].

#### 3.1.2. Population with Alzheimer’s Disease

**Zavecz and colleagues** [78] delved into the role of NREM sleep, particularly deep NREM SWS, in the development of CR within the context of AD pathology. Their hypothesis posited that the quality of NREM SWS, as indicated by SWA, might offer a compensatory mechanism against memory impairment induced by high AD pathology burden.

The study enrolled cognitively normal old adults, classified as β-amyloid positive (Aβ+) and β-amyloid negative (Aβ−). Methodologically, the research utilized positron emission tomography (PET) scanning to quantify Aβ levels, polysomnography (PSG) recordings for assessing SWA during NREM sleep, structural MRI scans to evaluate gray matter atrophy, and a hippocampal-dependent face–name learning task, which is sensitive to the effects of age and sleep. Additional variables such as education level and physical activity were considered as “traditional reserve builders”.

SWS is acknowledged for its role in enhancing learning and memory in healthy adults [66,79,80,81]. Multiple linear regression was employed to analyze the data. Results revealed that participants from both the Aβ+ and Aβ− groups who had higher quantity and quality of SWS performed better on memory tests. Moreover, NREM SWA significantly moderated the impact of Aβ status on memory function, suggesting enhanced memory performance in individuals with high Aβ burden, implying a compensatory function against cognitive decline. Furthermore, the CR function of NREM SWA remained significant even after controlling for other established CR builders such as education and physical activity, underscoring sleep as an independent contributor to CR development [78].

**Table 2:** Summary of reviewed studies focusing on sleep as a factor involved in CR.

References	Sleep Component/Disorder	Sleep Measure	Cognitive Function	Cognitive Function Measure	Reserve Measure	Results
Zijlmans et al. (2023) [22]	24 h activity rhythms (sleep onset latency and sleep efficiency)	7-day actigraphy; Sleep diary; PSQI.	-	-	Cognitive battery assessment: Verbal memory (15-word verbal learning test) Attention and interference of automatic processes (Stroop test) Long-term memory (word fluency test) Processing speed (letter–digit substitution task) Fine motor skills (Purdue pegboard test) Education level Brain-MRI Education	↑
Zavecz et al. (2023) [78]	NREM SWS AD-related	PSG	Memory	Hippocampal-dependent face–name learning task	Brain-MRI Education Physical activity	↑

Notes: For each reference the table shows: sleep component/disorder, cognitive function, and cognitive reserve and relative measures investigated. In results, upward arrow indicates sleep as a factor contributing to CR. Abbreviations: NREM: Non-rapid eye movement; SWS: Slow-wave sleep; AD: Alzheimer’s disease; PSQI: Pittsburgh Sleep Quality Index Questionnaire; PSG: Polysomnography; Brain-MRI: Brain-magnetic resonance imaging.

### 3.2. Cognitive Reserve as a Moderator in the Relationship between Sleep and Cognitive Functions

Nine articles examined CR as a potential mediator in the relationship between both physiological and pathological sleep patterns and cognitive functions. Out of these nine articles, four focused on a healthy elderly population [69,70,71,72], four focused on a population with sleep disorders [73,74,75,76], and one article focused on a pathological population with sleep disorders and another pathology, such as Parkinson’s disease (PD) [77] (see Table 3).

#### 3.2.1. Healthy Population

**Zimmerman et al.** [69] aimed to examine the relationship between sleep onset/maintenance difficulties (SO/MD) and cognitive functions in nondemented old adults, exploring the role of CR. Particularly, the study aimed to investigate how educational attainment might influence this relationship. The authors hypothesized that SO/MD would negatively impact cognitive functions such as attention, executive functions, and memory, and that individuals with lower education might be more vulnerable to these effects.

The research utilized a community-based sample. General mental status was assessed with the Blessed Information Memory-Concentration test. Participants completed a neuropsychological battery that assessed verbal memory, executive functions, and attention (for more details see Table 3). Additionally, they filled out a sleep questionnaire, the Medical Outcomes Study sleep scale (MOS-SS), to evaluate sleep difficulties. In the study, education was considered as a proxy for CR. The mean education level of the sample was 14.5 years, with individuals having 12 or fewer years of education designated as “lower education” and those with 13 or more years designated as “higher education”. General linear models were performed with cognitive performance as the dependent variable and SO/MD (present or absent) and education as between-subjects factors. Age, ethnicity, gender, depression, and cardiovascular comorbidities were included as covariates.

The results revealed that SO/MD difficulties are prevalent among older adults and have a detrimental effect on cognitive functions, particularly language fluency. Furthermore, older adults with lower levels of education and SO/MD performed poorer on language fluency tests compared to those with higher education levels. This result suggests that education can mitigate the impact of sleep difficulties on certain cognitive abilities, thereby implying that CR could offer protective effects against the cognitive impact of sleep difficulties [69].

**Parker and colleagues** [70] aimed to investigate the interplay between sleep quality, CR, and executive functions in older adults. The study explored how sleep disturbances, including the frequency and duration of awakenings, contribute to age-related declines in executive function. The authors hypothesized that individuals with higher CR might demonstrate less susceptibility to the negative effects of poor sleep quality on their executive function performance.

Community-dwelling older adults were recruited. Sleep was assessed through actigraphic monitoring and the Consensus Sleep Diary, which allowed cross-referencing of data obtained, respectively, from a physiological and a subjective method. Executive functions were assessed by analyzing nine measures. These factors were informed by Fisk and Sharp’s four-factor model to comprehensively measure inhibition, shifting, working memory, and generativity (for more details see Table 3). CR was estimated from the total years of full-time education.

Simultaneous regression and post hoc moderated mediation analyses were conducted to test whether CR compensates for sleep-related deficits in executive function. The data revealed that poorer sleep quality, characterized by frequent and prolonged awakenings, was associated with a decline in executive functions, particularly in individuals with lower CR. Conversely, higher education levels appeared to mitigate the negative impact of sleep disturbances on executive functions [70].

**Yeh et al.** [71] aimed to investigate the relationship between episodic memory and sleep–wake disturbances in old adults, by using both objective and perceived measures, and to examine CR as a potential moderator of this relationship.

Healthy older adults underwent sleep and memory assessments. Sleep–wake disturbances were measured using both objective and subjective components: objective assessment involved a 7-day actigraphy, while subjective measures involved completing the PSQI and the Epworth Sleepiness Scale questionnaire (ESS). CR was measured using the Wide Range Achievement Test 4-Reading subtest (WRAT-4-R) [82].

The analyses included Pearson’s correlation coefficients and hierarchical multiple regression. Results indicated that objectively measured sleep disruption (such as length of wakefulness during sleep, sleep disruption, and total sleep time) was associated with poorer episodic memory and learning, whereas greater perceived daytime sleepiness was linked to better episodic memory. Conversely, lower perceived daytime sleepiness was significantly correlated with poorer learning and delayed recall. CR did not moderate the relationship between sleep–wake disturbances and episodic memory. Specifically, while greater CR was associated with better delayed recall of episodic memory, it did not influence the impact of sleep–wake disturbances on episodic memory in this sample [71].

**Ourry et al.** [72] aimed to investigate the effect of CR on the association between SWS and cognition in older adults. Previous research has established associations between SWS and executive functions and memory processes. Moreover, SWS is known to decline with aging [19,83,84]. Accordingly, the study aimed to understand how CR proxies, assessed throughout the lifespan, might moderate the impact of sleep quality and changes on cognitive performance. This investigation is particularly relevant in the context of aging, where sleep disturbances can have various behavioral consequences.

Community-dwelling older adults underwent PSG and neuropsychological evaluation focusing particularly on memory and executive functions. Participants also completed a series of questionnaires to assess total CR and reserve across different life stages (early, mid, and late life), including the Cognitive Activities Questionnaire (CAQ) and the Lifetime of Experiences Questionnaire (LEQ), along with indicating years of education. The authors hypothesized that individuals with greater CR might show greater resistance to sleep disturbances. Composite scores for both executive functions and memory, particularly episodic memory, were derived from multiple assessments. Specifically, the executive functions score was based on four tests, while the episodic memory score was based on two tests (for more details see Table 3).

The authors investigated whether CR proxies (engagement in complex mental activities throughout life) moderate the association between SWS and cognition. Additionally, exploratory analyses were conducted to determine if there is a specific life stage during which the development of CR might be more critical in mitigating the effects of age-related sleep changes. Separate multiple linear regression analyses were conducted, revealing a positive, albeit not robust, association between the proportion of SWS and episodic memory, with no significant association found with executive functions. Furthermore, individuals with higher CR (as indicated with CAQ and LEQ scores) maintained better memory performance even when the percentage of SWS was low, whereas those with lower CR and less SWS demonstrated poorer cognitive performance. Finally, the association between SWS and memory was stronger when cognitive activities were accumulated during middle age [72].

#### 3.2.2. Population with Sleep Disorders

**Alchanatis et al.** [73] aimed to explore the relationship between OSA syndrome-related cognitive deficits in specific domains and the overall intellectual function of patients with OSA, investigating whether higher intelligence might protect against cognitive decline caused by OSA, presumably due to increased CR.

The sample consisted of OSA patients and healthy individuals. Participants underwent PSG and completed the ESS questionnaire to evaluate daytime sleepiness. Additionally, they participated in an attention/alertness battery test. Participants also completed the Raven’s Progressive Matrices intelligence test (RPM). Subsequently, based on the RPM Intelligence Quotient (IQ) score, considered as a proxy of CR, participants were divided into high-intelligence patients, high-intelligence controls, normal-intelligence patients, and normal-intelligence controls.

OSA patients underwent Continuous Positive Airway Pressure (CPAP) treatment for one year, but we will only discuss the part before treatment that is relevant to our review questions. Mann–Whitney U-test analyses showed that, before treatment, the high-intelligence patient group had similar results in attention/alertness tests compared to the high-intelligence control group. Conversely, the normal-intelligence patient group performed worse across all tests compared with the normal-intelligence control group. Therefore, OSA patients with higher intelligence did not show attention/alertness deficits compared to the high-intelligence control group, suggesting a protective effect of higher intellectual functioning against cognitive decline caused by OSA [73].

**Olaithe et al.** [74] aimed to explore the heterogeneity in cognitive dysfunction among individuals with OSA by identifying distinct cognitive profiles and examining how these profiles relate to other features of OSA. They also explored how CR and other variables can affect cognitive function in OSA patients.

The authors recruited two samples of individuals with untreated moderate to severe OSA from a sleep clinic and from the community, aged between 40 and 65. Participants underwent PSG and ApneaLink (ResMed Corporation, Poway, Calif), a single-channel recording device that measures airflow during sleep, to assess sleep recording details. Daytime sleepiness was evaluated using the ESS questionnaire. Additionally, cognitive functions were assessed using a cognitive battery that measures important aspects of attention, short-term memory, and episodic long-term memory. The National Adult Reading Test was used to determine the CR index.

Latent profile analysis was conducted on the above-mentioned cognitive function assessment to classify individuals with OSA into cognitive performance profiles. This technique identifies cognitive profiles based on scores across a set of indicator variables. Three cognitive profiles were identified: strong thinkers, inattentive thinkers, and slow but accurate thinkers. Only the strong thinkers showed a high CR. In the clinic sample, a higher CR moderated the impact of average oxygen saturation leading to better overall cognitive performance. However, CR did not influence cognitive profile membership in the community sample. These findings suggest that individual differences in CR and nocturnal oxygen saturation affect cognition in individuals with OSA [74].

**Hlaing et al.** [75] aimed to examine the interaction between educational level and OSA on cognitive functions, including verbal fluency, psychomotor vigilance, executive functions, visuospatial ability, and attention span in middle-aged and older patients with untreated OSA. 

The sample included healthy controls and untreated OSA patients, ranging in ages from 40 to 92 years. Education was considered as a proxy for CR, with OSA patients having a mean education level of 15 years and the healthy control group averaging 18 years of education. Participants underwent neuropsychological assessment to evaluate language, attention, executive functions, and visuospatial abilities (for more details see Table 3). Subjective sleep and health measures were assessed through the PSG, ESS, PSQI, and Morningness–Eveningness Questionnaire. 

Multiple regression was conducted using the backward method. Education was found to mitigate deficits caused by OSA in certain functions, such as semantic fluency and visuospatial abilities, purportedly due to increased CR. However, for other domains like executive functions and attention, higher education levels correlated with better performance irrespective of the presence of the disease. The study revealed that, particularly in tasks with higher cognitive demand, education serves as a protective factor for OSA patients, which outperformed both OSA patients and healthy controls with fewer years of education. Overall, these results indicate how education can contribute to mitigate some of the cognitive deficits associated with OSA, highlighting its role in maintaining cognitive health in middle-aged and older adults [75].

**D’Este et al.** [76] aimed to investigate the impact of CR on cognitive performance in patients with iRBD. The study explored how different levels of CR might influence the timing of cognitive decline and phenoconversion in iRBD patients, potentially contributing to the variability in progression among these individuals.

The authors recruited iRBD patients with an average age of 66.38 years. Participants underwent a comprehensive clinical evaluation, including neurological examination, video-PSG exam, motor and non-motor symptoms questionnaires, and the CR Index questionnaire (CRIq), to evaluate CR throughout the lifespan. Neuropsychological assessment was conducted to evaluate general cognitive function, language, memory, executive functions, and visual–spatial abilities (for more details see Table 3). The raw scores obtained in each neuropsychological test were adjusted for age, gender, and education, yielding adjusted scores for each patient. These corrected scores were evaluated based on a five-level scale indicating the level of cognitive impairment. Based on this neuropsychological evaluation, the presence of mild cognitive impairment (MCI) was assessed. 

Correlation analyses were performed between the corrected scores of neuropsychological tests and CRIq values. The data showed that patients with high levels of CR performed better in visuo-constructive and verbal memory functions, particularly in the recall of the Rey–Osterrieth complex figure test, compared to patients with a low level of CR. iRBD patients with higher levels of CR also showed a lower percentage of MCI. Taken together, these results suggest that a high CR might help iRBD patients in coping more effectively with cognitive decline related to neurodegenerative processes [76].

#### 3.2.3. Population with Parkinson’s Disease and Sleep Disorders

**Prete et al.** [77] aimed to examine how CR moderates the relationship between sleep difficulties and cognitive performance in PD patients. The researchers hypothesized that patients with lower CR levels would exhibit more pronounced cognitive deficits when experiencing sleep difficulties.

The study involved PD patients with an average age of 63.81 years. Researchers evaluated participants’ cognitive functions, CR levels, and various sleep-related symptoms. Subjective sleep information was recorded through completion of the REM sleep behavior disorder (RBD) Screening Questionnaire, PSQI, and ESS questionnaire. General cognitive functions were assessed using the Telephone-Global Examination of Mental State. CR was assessed through completion of the CRIq, to evaluate CR throughout the lifespan.

Correlation results confirmed a significant association between higher RBD symptoms and lower cognitive performance, particularly in executive functions, among PD patients with lower CR levels. Overall, CR appears to act as a protective factor against cognitive decline in PD patients with sleep difficulties. Therefore, PD patients with low-to-medium CR levels may face an increased risk of cognitive deficits in the presence of sleep disturbances [77].

**Table 3:** Summary of reviewed studies focusing on CR as mediator between sleep and cognitive functions.

Reference	Sleep Component/Disorder	Sleep Measure	Cognitive Function	Cognitive Function Measure	Reserve Measure	Results
Zimmerman et al. (2012) [69]	SO/MD difficulties	MOS-SS.	Verbal memory	Free and Cued Selective Reminding Test	Education	↑ language fluency
			Executive functions	Trail Making Test-Part B, Category Fluency; Letter Fluency Tests		- Executive functions
			Attention	Digit Span Subtest from WAIS-III; Trail Making Test-Part A		- Attention
Parker et al. (2020) [70]	Sleep quality (frequency and duration of awakenings)	Actigraphy; Consensus; Sleep Diary.	Inhibition	Anti-Saccades and Flanker tests;	Education	↑ Executive functions
			Shifting	NIH Examiner Set-Shifting Subtest; Trail Making Test-Part B; Verbal Fluency Category Switching Subtest from the Delis–Kaplan Executive Function System;		
			Working memory	Digit Span Backwards from WAIS-III;		
				Dual Task Subtest of the Test of Everyday Attention		
			Generativity	Controlled Oral Word Association Test; the Action Fluency Task		
Yeh et al. (2021) [71]	Sleep–wake disturbances	7-day actigraphy; PSQI; ESS questionnaire.	Episodic memory	Hopkins Verbal Learning Test-Revised	Wide Range Achievement Test 4-Reading subtest	- Episodic memory
Ourry et al. (2023) [72]	SWS	PSG	Executive functions	Digit Span Backward; Trail Making Test-Part B; Stroop Interference Test; Letter Fluency;	CAQ Questionnaire; LEQ Questionnaire.	- Executive functions
			Memory	The California Verbal Learning Test; Wechsler Memory Scale IV Logical Memory, Story B (WMS IV)		↑ Memory
Alchanatis et al. (2005) [73]	OSA	PSG; ESS questionnaire.	Attention/ alertness	Vienna Test System	RPM IQ	↑ Attention/Alertness
Olaithe et al. (2020) [74]	OSA	PSG; ApneaLink; ESS questionnaire.	Attention Short-term memory Episodic long-term memory	Cognitive Drug Research System	National Adult Reading Test	↑ Attention (only in the clinical sample) ↑ Memory (only in the clinic sample)
Hlaing et al. (2021) [75]	OSA	PSG; ESS questionnaire; PSQI; Morningness–Eveningness Questionnaire.	Language	Semantic and Phonemic Fluency Tests;	Education	↑ Language
			Attention	Psychomotor Vigilance Task;		↑ Attention (regardless of OSA)
			Visuospatial abilities	WAIS-III Block Design;		↑ Visuospatial abilities
			Executive functions	Wisconsin Card Sorting Test, WAIS-III digit span		↑ Executive functions (regardless of OSA)
D’Este et al. (2023) [76]	iRBD	PSG.	General cognitive function	Mini Mental State Examination	CRIq	- Cognitive functions
			Language	Token Test; Semantic and Phonemic Verbal Fluency Tests; CAGI Oral Denomination Test;		- Language
			Memory	Digit-Span Test; Corsi Block-Tapping Test; Rey Auditory Verbal Learning Test; Recall of the Rey Osterrieth Complex Figure;		↑ Verbal memory functions
			Executive functions	RPM Task; Attentive Matrices Test		- Executive functions
			Visuospatial abilities	Copy Rey Osterrieth Complex Figure		↑ Visuo-constructive functions
Prete et al. (2023) [77]	Sleep difficulties PD-related	RBD Screening Questionnaire; PSQI; ESS questionnaire.	General cognitive functions	Telephone-Global Examination of Mental State	CRIq	↑ Cognitive functions

Notes: For each reference the table shows: sleep component/disorder, cognitive function, and cognitive reserve and relative measures investigated. In results, upward arrow indicates a protective effect of CR in the relationship between sleep and cognitive function, while dash indicates no significant CR mediation in the relationship between sleep and cognitive function. Abbreviations: SWS: slow-wave sleep; SO/MD: sleep onset/maintenance; OSA: obstructive sleep apnea; iRBD: isolated rapid eye movement sleep behavior disorder; PD: Parkinson’s disease; PSG: polysomnography; PSQI: Pittsburgh Sleep Quality Index Questionnaire; ESS: Epworth Sleepiness Scale Questionnaire; MOS-SS: Medical Outcomes Study sleep scale; RBD: REM sleep behavior disorder; WAIS-III: Wechsler Adult Intelligence Scale-III; CAGI: Italian battery for the assessment of semantic memory disorders; RPM: Raven’s progressive matrices; CAQ: cognitive activities questionnaire; LEQ: lifetime of experiences questionnaire; CRIq: cognitive reserve index questionnaire; IQ: Intelligence Quotient.

## 4. Discussion

**Figure 2:**
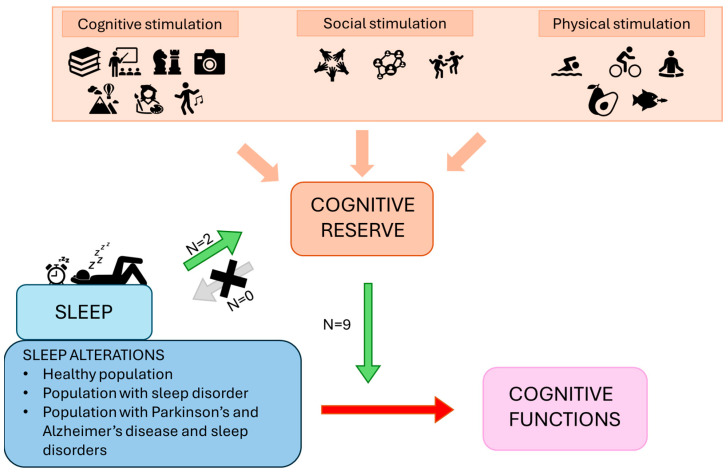
The panel represents a summary of the framework and results proposed. The green arrow represents the promoting effects of sleep on reserve, as well as the protective and ameliorative moderating effects of reserve on the relationship between sleep alterations and cognitive functions. The grey arrow suggests a lack of research investigating the role of CR as a protective or enhancing factor on sleep. The red arrow shows the detrimental effects of sleep alterations on cognition.

The aim of the present review was to investigate the relationship between sleep and BR/CR/NR in humans, specifically in relation to cognitive functions. It focuses on understanding the bidirectional influence these two components may exert on each other by analyzing the available literature connecting them. Table 2 and Table 3 detail the studies included in the analysis, while Figure 2 provides a summary panel depicting the evidence synthesized in this narrative review.

Overall, the evidence gathered regarding sleep and CR suggests a dearth of research investigating CR’s role as a protective or enhancing factor for sleep. However, two scientific studies were identified that examined sleep’s contribution to CR [22,78]. In contrast, most of the studies in the literature aimed to investigate CR as a moderator in the relationship between sleep, both physiological and pathological, and cognitive functions. Among the nine studies in this category, eight provided evidence indicating that CR exerts a protective influence on cognitive functions. This protective effect was observed in both healthy individuals and those with pathological conditions, regardless of whether they experienced sleep disturbances such as OSA [73,74,75], iRBD/RBD [76,77], or physiological changes in sleep architecture such as decreased SWS [72] or alterations in sleep duration and efficiency [69,70]. Conversely, only one study reported that CR did not play a significant role in mitigating the impact of sleep–wake disturbances on memory, specifically episodic memory [71]. Surprisingly, no study to date has investigated the potential protective influence of CR in patients with insomnia disorder, a highly prevalent condition associated with adverse health outcomes.

Therefore, the results of these studies align with the BR/CR/NR hypothesis [1,7,14,85], emphasizing the protective impact and beneficial effects of reserve on cognitive functions [6,75,77,86,87,88,89]. Additionally, sleep, acknowledged as modifiable lifestyle factor [15,90], plays a critical role in promoting CR development and enhancing cognition while mitigating brain changes during both physiological and pathological aging [22,78,91,92,93].

This review provides a compelling framework by delving deeper than merely illustrating how CR attenuates and safeguards brain and cognitive functions during both physiological and pathological aging (e.g., neurodegenerative diseases), phases characterized by cognitive decline. Instead, it demonstrates how CR actively participates in compensating for alterations in sleep components due to advancing age in healthy adults, as well as in sleep disorders affecting both healthy and pathological individuals [69,70,72,73,74,75,76,77]. Furthermore, it emphasizes how specific sleep components (e.g., SWS) or sleep activity rhythms (e.g., sleep onset latency or sleep efficiency) could act as CR factors, providing resilience against impaired conditions [22,72,78].

An important aspect to consider is that cognitive abilities, preserved and strengthened through experience, can be maintained as a reserve and be accessed when needed, especially in tasks requiring high cognitive demand [75]. This applies not only to neurodegenerative or age-related disorders [8] but also to other clinical conditions such as sleep disruption or diseases [75].

Another significant observation from the reviewed articles is the substantial heterogeneity in the methods employed to investigate the topic. Authors have approached the examination of specific sleep components and cognitive functions in various ways. Additionally, it is important to note that CR is a latent construct and cannot be directly measured; therefore, it relies on proxy indicators [94]. However, most of the studies included in this review utilize different single proxy measures of CR, such as education level [69,70,75], IQ [73], CRIq [76,77], WRAT-4-R [71], or the National Adult Reading Test [74].

The heterogeneity arising from the various approaches using single proxy measures for CR underscores the need for adopting a definitive and standardized composite measure of reserve [88,95], as advocated by Stern et al. [12]. Such a composite measure is crucial for reducing inconsistencies among study results and enabling direct comparisons between investigations of specific sleep components and their effects on different cognitive domains.

Another limitation in this context is the absence of studies explicitly aimed at investigating NR or BR, which would include both functional and structural measurements of the brain. Among the articles reviewed, only two reported on brain structural measurement via the use of brain-MRI [22,78]. However, the authors used this structural correlate to provide information on the pathological [78] and physiological [22] condition of participants, rather than as a direct measure of BR or NR. Indeed, integrating a structural brain evaluation into the quantification of CR proxies could be an important consideration for future studies, enabling a more robust correlation and precise measurement of NR.

Lastly, the experimental samples enrolled in the studies exhibit heterogeneity in the age range considered, and there are no studies investigating the relationship between sleep and BR/CR/NR in a young population. Future studies could benefit from exploring the bidirectional relationship between these components in adolescent and young adult populations. Additionally, focusing on a more restricted age range could help to better understand the effects of CR on sleep and vice versa.

## 5. Conclusions

The findings of the present review support that both CR and sleep are critical factors in preventing and treating cognitive decline in both physiological and pathological aging (see Figure 2). Sleep, as a modifiable lifestyle factor, holds promise as an intervention to preserve cognitive functions, with SWS being a particularly important therapeutic target. Additionally, CR serves as a protective factor against cognitive decline, benefiting not only individuals with age-related pathological conditions but also those with sleep difficulties and disorders such as OSA or iRBD/RBD. These findings underscore the importance of considering individual differences in CR when examining the impact of sleep on cognitive functions. Overall, addressing both CR and sleep offers significant potential for interventions aimed at delaying cognitive decline and promoting healthy brain aging.

Future research should further enhance our understanding of the relationship between BR/CR/NR and sleep, focusing on how these factors influence each other. Investigating the role of EEG activities, not only in SWS but also in parameters such as hippocampal ripples and thalamic spindles, would provide valuable insights into their effects on cognitive performance and, in association, on CR. Additionally, employing composite measures to better assess CR and expanding research to include young populations are essential steps. These efforts could significantly contribute to developing actions to prevent cognitive decline in both healthy and pathological populations, as well as in individuals with sleep-related conditions.

## Figures and Tables

**Table 1:** Descriptive characteristics of the included studies.

Reference	Population	Age Range (Years)	Mean Age (Years)	Sex	Sample Size (N)
Zimmerman et al. (2012) [69]				SO/MD L.E. F 67.2%	549
	Healthy	71–97	79.7	SO/MD H.E. F 60%	
				No SO/MD L.E. F 59%	
				No SO/MD H.E F 62.4%	
Parker et al. (2020) [70]	Healthy	55–93	71.7	N/A	184
Yeh et al. (2021) [71]	Healthy	60–88	69.9	F 75.8%	62
Ourry et al. (2023) [72]	Healthy	65–84	69.4	F 61.5%	135
Alchanatis e al. (2005) [73]	OSA	N/A	N/A	N/A	83
Olaithe et al. (2020) [74]	OSA	40–65	SCS 53.94	SCS M N = 64; 52.9%	519
			CS 60	CS M N = 245; 61%	
Hlaing et al. (2021) [75]	OSA	OSA 40–92	OSA 54.82	OSA F 56.5%	109
		CTRL 40–81	CTRL 56.60	CTRL F 80.9%	
D’Este et al. (2023) [76]	iRBD	50–78	66.38	F 80%	55
Prete et al. (2023) [77]	PD	N/A	63.81	F N = 60.4%	43
Zijlmans et al. (2023) [22]	Healthy	58–72	65.0	F 51.3%	1002
Zavecz et al. (2023) [78]	AD	68–72	Aβ+ 75.97	F Aβ+ 68%	62
			Aβ− 75.26	F Aβ− 55%	

Notes: Abbreviations: OSA: obstructive sleep apnea; iRBD: isolated rapid eye movement sleep behavior disorder; PD: Parkinson’s disease; AD: Alzheimer’s disease; N/A: not applicable; CTRL: control group; Aβ+: β-amyloid positive; Aβ−: β-amyloid negative; SO/MD L.E F: sleep onset/maintenance difficulties low-education female; SO/MD H.E. F: sleep onset/maintenance difficulties high-education female; No SO/MD L.E. F: No sleep onset/maintenance difficulties low-education female; No SO/MD H.E F: No sleep onset/maintenance difficulties high education female; F: female; M: male; N: number; SCS M: sleep clinic sample male; CS M: Community sample male.

## Data Availability

The original contributions presented in this study are included in the article, further inquiries can be directed to the corresponding author.
